# Clinical and Economic Value of Rapid Microbiological Diagnostics in Bloodstream Infections: A State-of-the-Art Evidence Review with Emphasis on PCR and MALDI-TOF

**DOI:** 10.3390/microorganisms14050994

**Published:** 2026-04-28

**Authors:** Ralitsa Raycheva, Gergana Lengerova, Michael Petrov, Todor Kantardjiev

**Affiliations:** 1 Department of Social Medicine and Public Health, Medical University Plovdiv, 15A V. Aprilov Blvd., 4002 Plovdiv, Bulgaria; 2 Department of Medical Microbiology and Immunology “Prof. Dr. Elissay Yanev”, Medical University Plovdiv, 15A V. Aprilov Blvd., 4002 Plovdiv, Bulgaria; gergana.lengerova@mu-plovdiv.bg (G.L.); michael.petrov@mu-plovdiv.bg (M.P.); todorkantardjiev@gmail.com (T.K.); 3 Microbiology Laboratory, St. George University Hospital, 15A V. Aprilov Blvd., 4002 Plovdiv, Bulgaria; 4 Research Institute, Medical University Plovdiv, 15A V. Aprilov Blvd., 4002 Plovdiv, Bulgaria

**Keywords:** bloodstream infections, rapid microbiological diagnostics, antimicrobial stewardship, MALDI-TOF, PCR, cost-effectiveness, health economics, sepsis, hospital length of stay, antimicrobial resistance

## Abstract

Bloodstream infections (BSIs) are associated with substantial morbidity, mortality, and healthcare costs. Conventional diagnostics are limited by delayed results, often postponing appropriate antimicrobial therapy. This review aimed to evaluate the clinical and economic value of rapid microbiological diagnostics in BSI management. A state-of-the-art evidence synthesis was conducted using structured searches of PubMed/MEDLINE, Scopus, Web of Science, EconLit, and Google Scholar (2013–2025). Eligible studies included economic evaluations and clinical studies reporting downstream economic or resource-use outcomes. Screening and data extraction were performed by two reviewers, and findings were narratively synthesized. Fifty-nine studies were included. Rapid diagnostics consistently reduced time to pathogen identification and targeted therapy compared to conventional methods. Molecular platforms provided results within hours, while MALDI-TOF enabled identification within 30–60 min after culture positivity. Clinical benefits included earlier therapy optimization, reduced mortality, and shorter hospital stays, particularly when combined with antimicrobial stewardship programs (ASPs). Economic evaluations demonstrated improved cost-effectiveness, including reduced hospitalization, ICU utilization, and antimicrobial costs. MALDI-TOF with stewardship showed notable cost savings and improved outcomes. However, results varied depending on implementation context, infrastructure, and workflow integration. Rapid microbiological diagnostics offer significant clinical and economic benefits in BSI management, particularly when integrated with stewardship programs. Context-specific implementation is essential to maximize their value across healthcare systems.

## 1. Introduction

### 1.1. Global Burden of Bloodstream Infections (BSIs)

Rapid microbiological methods for the diagnosis and management of bloodstream infections are generally cost-effective; especially when integrated with antimicrobial stewardship programs. Multiple decision-analytic models and meta-analyses demonstrate that rapid diagnostic tests (RDTs)—such as polymerase chain reaction (PCR), matrix-assisted laser desorption/ionization time-of-flight mass spectrometry (MALDI-TOF), and multiplex molecular panels—reduce time to targeted therapy, length of hospital stay, and mortality, with the greatest benefit observed when combined with active antimicrobial stewardship interventions [1,2,3,4,5].

Economic analyses show that strategies like MALDI-TOF with stewardship can save up to USD 29,205 per quality-adjusted life year and avert one death per 14 patients tested, compared to conventional methods without stewardship. The probability of cost-effectiveness for rapid diagnostics with stewardship exceeds 80% in probabilistic models [1]. However, isolated rapid diagnostics without stewardship are less consistently cost-effective, and some randomized controlled trials (e.g., RAPIDO) suggest that adjunctive MALDI-TOF may not be cost-effective in all settings, though real-world implementation may differ [6].

Globally, bloodstream infections (BSIs) are a major cause of morbidity and mortality, with sepsis ranking among the leading causes of death and imposing substantial healthcare costs. Delays in appropriate therapy increase mortality risk hourly, underscoring the clinical and economic imperative for rapid diagnostics [5,7,8]. The burden is particularly high in resource-limited settings, where diagnostic delays and inappropriate empiric therapy contribute to poor outcomes and antimicrobial resistance [8].

### 1.2. Limitations of Traditional Microbiological Diagnostics (Time-to-Result Delays)

Rapid microbiological methods are cost-effective in the diagnosis of bloodstream infections primarily because they significantly reduce time-to-result compared to traditional blood culture-based diagnostics, which typically require 1–7 days for organism identification and susceptibility testing [7,9,10]. Delays inherent to conventional methods are associated with increased mortality, longer hospital stays, and higher healthcare costs, as every hour of delay in appropriate therapy increases the risk of adverse outcomes [7,11].

Rapid diagnostic tests (RDTs)—including molecular assays, MALDI-TOF, and multiplex PCR panels—can provide actionable results within 1–4 h, enabling earlier targeted antimicrobial therapy and improved patient outcomes [7,10,12]. Economic modeling demonstrates that the combination of rapid diagnostics with antimicrobial stewardship programs is the most cost-effective strategy, resulting in substantial savings per quality-adjusted life year and a high probability of cost-effectiveness in probabilistic analyses [1,2].

Limitations of traditional diagnostics—such as long turnaround times, reduced sensitivity in patients already on antibiotics, and inability to rapidly detect resistance markers—are directly addressed by rapid methods, which improve the timeliness and accuracy of pathogen identification [7,9,10]. The American Society for Microbiology recommends rapid tests combined with active communication to decrease time to targeted therapy and length of stay, supporting their clinical and economic utility in hospitalized patients with suspected BSI [12].

### 1.3. Emergence of Rapid Microbiological Identification (e.g., PCR, MALDI-TOF, Multiplex Panels)

Rapid microbiological methods such as PCR, MALDI-TOF, and multiplex panels are cost-effective for the identification of bloodstream infections, especially when integrated with antimicrobial stewardship programs. These technologies markedly reduce time-to-result compared to traditional blood cultures, enabling earlier optimization of antimicrobial therapy, which is associated with improved clinical outcomes and reduced healthcare costs [1,12,13,14,15,16].

Decision-analytic models and randomized controlled trials demonstrate that MALDI-TOF with stewardship yields substantial savings per quality-adjusted life year (QALY) and prevents more deaths compared to conventional methods. For example, MALDI-TOF with stewardship saves USD 29,205 per QALY and averts one death per 14 patients tested, while PCR-based methods and multiplex panels also show favorable incremental cost-effectiveness ratios [1,13,14,15]. Multiplex PCR panels, such as the BioFire FilmArray BCID, are highly sensitive and specific, and their use leads to earlier targeted therapy, shorter hospital stays, and lower overall costs, despite higher upfront laboratory expenses [13,14,16].

The American Society for Microbiology recommends rapid diagnostic tests combined with active communication to decrease time to targeted therapy and length of stay, supporting their cost-effectiveness in routine clinical practice [12]. However, the cost-effectiveness is maximized when these rapid methods are paired with stewardship interventions; without stewardship, the probability of cost-effectiveness is significantly lower [1,15].

Although a range of rapid diagnostic technologies is emerging, including loop-mediated isothermal amplification (LAMP), microarray-based platforms, and next-generation sequencing approaches, PCR-based assays and MALDI-TOF mass spectrometry have emerged as the most widely implemented rapid diagnostic technologies in the current global clinical microbiology landscape, particularly in high-income hospital settings. These platforms are increasingly integrated into antimicrobial stewardship workflows and real-time clinical decision-making processes, enabling earlier pathogen identification and more timely therapeutic optimization. Their widespread adoption in routine practice, combined with their predominant representation in the available clinical and economic evaluation literature, makes them central to understanding the real-world clinical and economic impact of rapid diagnostics and underpins their emphasis in the present review.

### 1.4. Rationale for Economic Evaluation: Improving Clinical Outcomes and Reducing Costs

The rationale for conducting an economic evaluation of rapid microbiological methods in the diagnosis and management of bloodstream infections is to determine whether these technologies improve clinical outcomes and reduce healthcare costs compared to conventional diagnostics.

Rapid methods such as PCR, MALDI-TOF, and multiplex panels significantly decrease time to pathogen identification, enabling earlier initiation of targeted antimicrobial therapy. This leads to reductions in mortality, length of hospital stay, and inappropriate antibiotic use, which are key drivers of healthcare costs in patients with bloodstream infections [2,3,12,17]. Economic models consistently show that rapid diagnostics, especially when combined with antimicrobial stewardship programs, are cost-effective—resulting in substantial savings per quality-adjusted life year and a high probability of cost-effectiveness in probabilistic analyses [1,5,7,14,18].

Traditional blood culture-based diagnostics are limited by long turnaround times (often 1–7 days), which delay appropriate therapy and increase the risk of adverse outcomes and resource utilization [4,7,8,9]. By contrast, rapid methods can provide actionable results within hours, directly impacting patient management and hospital resource allocation.

Therefore, economic evaluation is essential to justify the adoption of rapid diagnostics by quantifying their impact on both patient outcomes and healthcare expenditures, ensuring that investments in new technologies translate into meaningful clinical and financial benefits [1,2,12,14,18].

The objective of this narrative review is to critically examine the clinical and economic value of rapid microbiological diagnostic methods used in the diagnosis and management of bloodstream infections (BSIs), with a particular focus on their impact on patient outcomes, antimicrobial stewardship, and healthcare resource utilization. The review aims to synthesize and contextualize existing evidence on whether and under what conditions rapid diagnostic technologies deliver meaningful clinical benefits and represent cost-effective or cost-saving strategies compared with conventional blood culture–based workflows.

## 2. Materials and Methods

This review was designed as a state-of-the-art evidence synthesis, integrating structured literature identification with narrative thematic analysis. While incorporating key elements of systematic review methodology—such as predefined search strategies, explicit inclusion criteria, and dual-reviewer screening—the review does not aim to provide quantitative effect estimation or formal meta-analysis. Instead, its primary objective is to synthesize and contextualize heterogeneous clinical and economic evidence, identify key drivers of value, and examine implementation-relevant factors across diverse healthcare settings. This approach is consistent with guidance from the Joanna Briggs Institute for evidence synthesis in areas characterized by methodological heterogeneity and evolving evidence bases.

The decision to adopt a state-of-the-art evidence synthesis was based on three main considerations. First, economic evaluations of rapid diagnostic technologies for BSIs differ substantially in study design, diagnostic platforms, analytical perspective, time horizon, and outcome measures, limiting the feasibility of quantitative pooling or formal meta-analysis.

Second, the purpose of this review was not only to summarize cost-effectiveness results, but also to examine methodological approaches, drivers of economic value, and contextual factors, including antimicrobial stewardship integration and health system characteristics.

Third, the multidisciplinary and evolving nature of the literature—spanning clinical microbiology, infectious diseases, health economics, and hospital management—necessitated an integrative synthesis capable of capturing emerging technologies and real-world implementation issues beyond the scope of narrowly defined systematic reviews. This approach allows for a more comprehensive understanding of the policy relevance and implementation value of rapid diagnostics in BSI management.

This review was reported in accordance with the PRISMA 2020 guidelines where applicable. Given its state-of-the-art (narrative) design and the heterogeneity of the included studies, the review does not follow a full systematic review framework, and no quantitative synthesis was performed. A completed PRISMA 2020 checklist is provided in Appendix A.2 to enhance transparency of reporting; items not applicable to the narrative synthesis approach are indicated accordingly.

A PRISMA flow diagram was included to transparently present the study selection process. A PRISMA 2020 flow diagram for studies based on database and register searches only was used, as the search strategy did not include additional sources such as websites, organizational data, or citation tracking (https://www.prisma-statement.org/prisma-2020-flow-diagram (accessed on 3 February 2026)—PRISMA 2020 flow diagram for new systematic reviews, which included searches of databases and registers only).

### 2.1. Search Strategy

A structured literature search was conducted in five electronic databases: PubMed (MEDLINE), Scopus (Elsevier), Web of Science (Core Collection), EconLit and Google Scholar. Searches covered the period from 1 January 2013 to 30 November 2025.

The search strategy combined three main concept blocks using Boolean logic:

(1) Bloodstream infections and sepsis;

(2) Rapid microbiological diagnostics (e.g., molecular assays, multiplex PCR, MALDI-TOF, rapid phenotypic susceptibility testing); and

(3) Economic evaluation and resource utilization (e.g., cost-effectiveness, cost–utility, budget impact, length of stay).

Search syntax was adapted to the indexing structure of each database; the complete search strings, filters, and record yields are presented in Appendix A—Database Search Strategies (Table A1). Reference lists of included studies and relevant reviews were manually screened to ensure completeness. All retrieved citations were imported into Rayyan Platform (https://www.rayyan.ai/, accessed on 4 December 2025) for reference management and removal of duplicate records.

### 2.2. Inclusion and Exclusion Criteria

Studies were included if they met the following criteria:

Design: Peer-reviewed economic evaluations (cost-effectiveness, cost–utility, cost–benefit, or budget impact analyses), modeling studies (decision-analytic or simulation models), or clinical studies reporting downstream economic or resource-use outcomes.

Setting: Hospital or acute care settings, including intensive care and non-intensive care units.

Interventions: Rapid microbiological diagnostic methods used for BSI identification or susceptibility testing (e.g., PCR-based panels, MALDI-TOF, rapid phenotypic platforms), with or without integration into antimicrobial stewardship programs.

Perspective: Analyses conducted from a hospital, healthcare system, payer, or societal perspective.

Outcomes: Reported clinical outcomes (e.g., time to appropriate therapy, mortality, length of stay) and/or economic outcomes such as incremental cost-effectiveness ratios (ICERs), cost per quality-adjusted life year (QALY) gained, cost per life saved, or budget impact estimates.

Publication characteristics: English-language, peer-reviewed journal articles.

Studies were excluded if they:

(1) Did not report clinical or economic outcomes relevant to rapid diagnostics;

(2) Were editorials, commentaries, conference abstracts, or non-peer-reviewed publications;

(3) Focused exclusively on laboratory performance without downstream clinical or economic implications;

(4) Lacked quantitative outcome reporting; or

(5) Were unavailable in full text.

### 2.3. Screening and Data Extraction

Titles and abstracts were screened for relevance, followed by full-text assessment of eligible articles. Screening and data extraction were performed by two reviewers, with discrepancies resolved through discussion and consensus.

Extracted data included study setting, type of rapid diagnostic technology, integration with antimicrobial stewardship, study design, analytical framework, economic perspective, time horizon, and key clinical and economic outcomes (e.g., ICERs, QALYs gained, mortality reduction, length of stay). Additional variables captured included drivers of cost-effectiveness, such as reductions in time to targeted therapy, antimicrobial de-escalation, and ICU utilization.

### 2.4. Appraisal of Included Studies

To enhance transparency and interpretive validity, included studies were critically appraised qualitatively, drawing on key domains from the Consolidated Health Economic Evaluation Reporting Standards (CHEERS 2022) and, where applicable, the Philips checklist for model-based economic evaluations.

The appraisal focused on:

- Justification of study perspective and comparators;
- Appropriateness and transparency of analytical framework or model structure;
- Time horizon and discounting assumptions;
- Sources and relevance of cost and outcome data;
- Handling of uncertainty (deterministic and/or probabilistic sensitivity analyses);
- Consideration of antimicrobial stewardship and contextual health system factors;
- Validation or calibration of models, where applicable;
- Overall reporting quality and clarity.
- Given the narrative design, no formal scoring or exclusion based on quality thresholds was applied. Instead, methodological strengths and limitations were considered during synthesis and are reflected in the interpretation of results.

Consistent with state-of-the-art review methodology, studies were not excluded based on formal quality scoring; instead, methodological strengths and limitations were integrated narratively into the interpretation of findings.

Particular attention was given to the reporting of cost components, currency year, and analytical perspective, given their importance for interpreting transferability of economic findings.

No formal scoring or quantitative weighting of study quality was performed. This approach was chosen in line with methodological guidance discouraging the use of aggregate quality scores in economic evaluations, particularly in the presence of substantial heterogeneity. Instead, appraisal findings were used to inform the interpretation of results, with particular attention to key domains such as model assumptions, time horizon, handling of uncertainty, and transparency of reporting.

### 2.5. Evidence Synthesis

Findings were synthesized using a thematic narrative approach, integrating clinical and economic evidence across study designs, diagnostic platforms, and healthcare contexts. The synthesis aimed to identify consistent patterns, key drivers of economic value, sources of heterogeneity, and implementation-relevant insights, with particular emphasis on the role of antimicrobial stewardship and health system infrastructure in determining the real-world value of rapid microbiological diagnostics for bloodstream infections. Accordingly, the findings should be interpreted as a qualitative, integrative synthesis of the available evidence rather than as pooled quantitative estimates, and conclusions are intended to reflect patterns, consistencies, and contextual determinants rather than precise effect sizes.

Due to substantial heterogeneity in cost reporting, currency year, analytical perspective, and healthcare system context across included studies, no standardization of monetary values (e.g., inflation adjustment or currency conversion) was performed. Economic outcomes are therefore presented as reported in the original studies and interpreted within their respective study contexts. The synthesis explicitly accounted for methodological heterogeneity and variability in study quality. Findings were interpreted in light of identified strengths and limitations of the included studies, rather than assuming equal evidentiary weight across all sources.

Particular attention was given to distinguishing between studies evaluating standalone diagnostic performance and those assessing integrated diagnostic–stewardship interventions.

The retrieved literature was predominantly focused on PCR-based diagnostics and MALDI-TOF platforms, which therefore constitute the main analytical emphasis of this review.

## 3. Results

**Figure 1:**
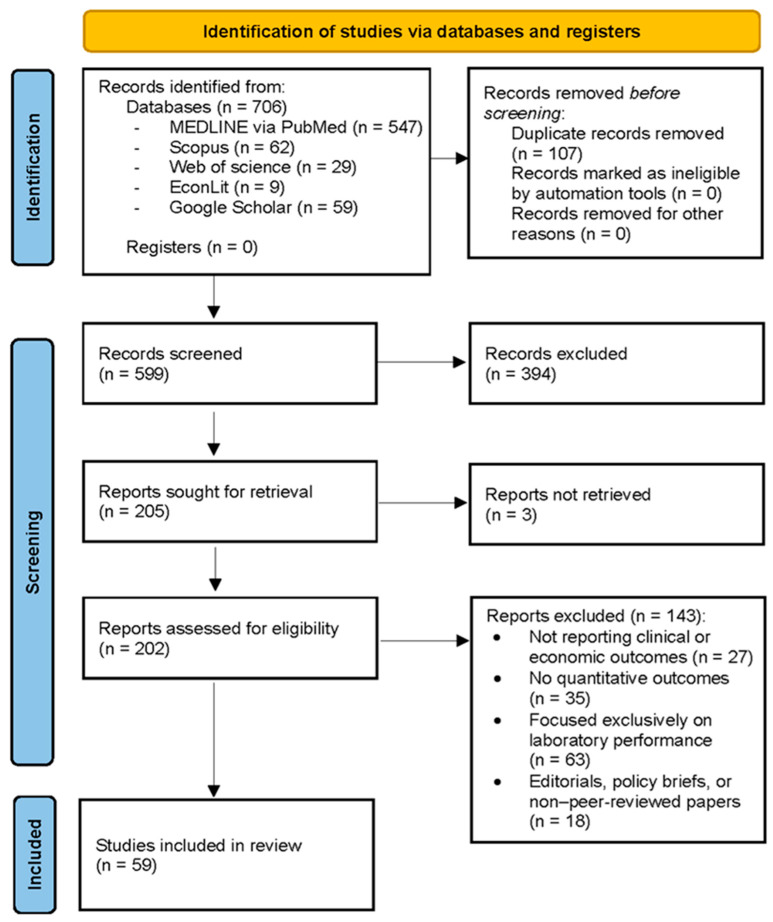
PRISMA diagram of the screening process.

The study selection process is summarized in the PRISMA 2020 flow diagram (Figure 1). A total of 706 records were identified through database searches, including MEDLINE via PubMed (*n* = 547), Scopus (*n* = 62), Web of Science (*n* = 29), EconLit (*n* = 9), and Google Scholar (*n* = 59). No additional records were identified through study registers. After removal of 107 duplicate records, 599 unique records were screened based on titles and abstracts. During the screening phase, 394 records were excluded for not meeting the predefined inclusion criteria. Subsequently, 205 reports were sought for full-text assessment, of which three could not be retrieved. The remaining 202 full-text articles were assessed for eligibility. Of these, 143 articles were excluded following full-text review. The main reasons for exclusion were absence of reported clinical or economic outcomes (*n* = 27), lack of quantitative results (*n* = 35), exclusive focus on laboratory or technical performance without downstream clinical or economic implications (*n* = 63), and publication types not meeting inclusion criteria, such as editorials, policy briefs, or non-peer-reviewed papers (*n* = 18). In total, 59 studies fulfilled all eligibility criteria and were included in the final synthesis.

Across included studies, the economic value of rapid microbiological diagnostics was strongly modified by stewardship integration, workflow organization, and clinical context (Table 1). The majority of the included studies evaluated PCR-based assays and MALDI-TOF platforms, reflecting the current concentration of clinical and economic evidence in this field.

**Table 1:** Determinants of clinical and economic value of rapid microbiological diagnostics in bloodstream infections.

Determinant	Direction of Effect on Value	Evidence Synthesis (Results-Level)	Key References
Diagnostic modality (PCR panels, MALDI-TOF, rapid AST)	Positive, modality-dependent	All major rapid modalities demonstrate potential economic value relative to conventional workflows when embedded in optimized systems	[1,6,14]
Reduction in time to targeted therapy	Strong positive	Earlier organism identification and susceptibility information consistently underpins downstream clinical and economic benefits	[2,3,17]
Integration with antimicrobial stewardship	Critical positive modifier	Stewardship determines whether diagnostic gains translate into therapeutic optimization and resource savings	[1,19,20]
Laboratory workflow and operating hours	Context-dependent	Limited laboratory availability attenuates the time-to-result advantage and reduces economic benefit	[21,22]
Disease severity/ICU case mix	Amplifying	Greater economic value observed in severe sepsis and high-acuity populations due to higher avoidable costs	[23,24]
Baseline antimicrobial resistance prevalence	Amplifying	Higher resistance increases value of early optimization and avoidance of inappropriate therapy	[2,25]
Test cost and reimbursement context	Constraining	High assay costs or lack of reimbursement reduce probability of cost-effectiveness despite clinical benefit	[6,26]
Health system resources (LMIC settings)	Variable/limiting	Infrastructure and stewardship capacity constrain economic value despite potential clinical benefit	[27,28]

Rapid microbiological methods for bloodstream infections are classified into four main categories: phenotypic rapid tests (accelerated culture-based methods), genotypic or molecular methods, mass spectrometry (MALDI-TOF), and point-of-care diagnostic platforms.

Phenotypic rapid tests accelerate traditional culture-based identification and susceptibility testing. Examples include direct-from-blood-culture identification using methods like the Accelerate Pheno system, which combines morphokinetic cellular analysis with fluorescence in situ hybridization to provide organism ID and phenotypic antimicrobial susceptibility within hours of blood culture positivity. These methods can also include rapid direct antimicrobial susceptibility testing platforms such as dRAST and VITEK REVEAL, which provide results within 4–8 h after blood culture positivity [21,29,30].

Genotypic or molecular methods detect pathogen DNA or RNA and resistance genes directly from positive blood cultures or, in some cases, directly from whole blood. Technologies include multiplex PCR panels (e.g., BioFire FilmArray BCID2, Luminex VERIGENE, Seegene Magicplex), microarray-based assays (e.g., Verigene), and T2 magnetic resonance (T2MR) panels. These methods can identify a broad range of pathogens and key resistance markers within 1–4 h, with high sensitivity and specificity [4,7,9,21,29,31].

Mass spectrometry (MALDI-TOF), such as the Bruker Sepsityper or FLAT MS, enables rapid identification of bacteria and yeast directly from positive blood cultures by analyzing protein spectra. MALDI-TOF can provide species-level identification within 30–60 min after blood culture positivity, with high accuracy for monomicrobial samples [21,29,30,32,33].

Point-of-care diagnostic platforms are emerging technologies designed for rapid, near-patient testing. These include cartridge-based molecular assays and isothermal amplification platforms, which can deliver results in under an hour and are being developed for direct-from-blood or direct-from-blood-culture applications. While not yet as widely adopted as laboratory-based systems, these platforms aim to further reduce time-to-result and facilitate earlier clinical decision-making [9,21,29].

The American Society for Microbiology and the Infectious Diseases Society of America recommend the use of rapid diagnostic tests, particularly when combined with active communication and stewardship, to decrease time to targeted therapy and hospital length of stay in patients with bloodstream infections [12,34].

### 3.1. Indicators of Performance: Time-to-Identification, Sensitivity, Specificity

Time-to-identification for rapid methods is typically 1–4 h for most molecular platforms (e.g., BioFire FilmArray BCID2, T2Bacteria, InfectID-BSI, RaPID/BSI), and 30–60 min for mass spectrometry-based methods such as MALDI-TOF when performed directly from positive blood cultures. This is a substantial reduction compared to conventional blood culture workflows, which require 16–72 h for identification and susceptibility results [7,12,30,32,33,35,36,37].

Sensitivity for rapid molecular tests and multiplex panels is generally 92–99% for common Gram-negative and Gram-positive bacteria, and yeast compared to standard-of-care phenotypic methods. For direct-from-blood molecular assays, sensitivity may be slightly lower (e.g., 76–90%) but is often superior in patients receiving antibiotics or with fastidious organisms [7,31,35,36,37,38,39]. MALDI-TOF platforms typically achieve 80–98% sensitivity for monomicrobial cultures, with lower performance in polymicrobial or rare pathogens [30,32,33].

Specificity for these rapid methods is consistently 90–100%, with most platforms reporting values at the upper end of this range for targeted organisms. Negative predictive values are also high (often > 99%), supporting their utility as rule-out tests [7,31,35,36,37,39].

The integration of rapid diagnostic tests (RDTs) with stewardship leads to faster time to optimal therapy (reductions of 18–29 h), lower mortality (odds ratio for death 0.72–0.78), and shorter hospital stays (odds ratio for reduced length of stay 0.91) [2,17]. These benefits are not observed with RDTs alone or stewardship alone; the combination is essential for clinical impact [2,17].

Rapid molecular and phenotypic platforms consistently decrease time to targeted therapy and facilitate more frequent and timely antibiotic modifications, including escalation for resistant organisms and de-escalation to narrow-spectrum agents [40,41,42,43,44]. This results in improved antibiotic utilization and, in some studies, reduced antimicrobial days of therapy [44,45]. The American Society for Microbiology recommends rapid tests with active communication to decrease time to targeted therapy and length of stay in hospitalized patients with bloodstream infections [12].

While some randomized trials show no difference in mortality or length of stay with rapid susceptibility testing alone, real-world studies and meta-analyses demonstrate that the clinical impact is maximized when rapid diagnostics are embedded within stewardship programs [2,3,17,46].

### 3.2. Economic Evidence and Evaluation Approaches

The types of economic evaluations reported in the assessment of rapid microbiological methods for bloodstream infection diagnosis include most commonly cost-effectiveness analysis, cost–utility analysis, cost–benefit analysis, and budget impact evaluations (Table 2).

Cost-effectiveness analysis is the most frequently used approach, comparing the incremental costs and clinical outcomes (such as deaths averted or time to appropriate therapy) of rapid diagnostic tests versus conventional methods, often expressed as incremental cost-effectiveness ratios (ICERs) [1,6,14,23,24]. Cost–utility analysis is also commonly performed, using quality-adjusted life years (QALYs) as the outcome measure to assess the value of rapid diagnostics in terms of both cost and patient-centered benefit [1,14,26]. Cost–benefit analysis is less common but has been used to quantify the net monetary benefit of rapid methods, particularly in randomized controlled trial settings [6]. Budget impact evaluations are performed to estimate the financial implications of adopting rapid diagnostics at the institutional or health system level, considering factors such as assay cost, length of stay, and resource utilization [14,23,24]. These analyses consistently show that rapid microbiological methods, especially when combined with antimicrobial stewardship programs, are cost-effective or cost-saving, with the greatest impact on mortality, length of stay, and overall healthcare expenditures [1,2,14,23].

Common economic outcomes reported in the evaluation of rapid microbiological methods for bloodstream infection diagnosis include cost per life saved, cost per quality-adjusted life year (QALY) gained, reduction in hospital length of stay, avoided antimicrobial costs, avoided intensive care unit (ICU) admissions, and impact on antimicrobial resistance.

Cost per life saved and cost per QALY gained are frequently used metrics. For example, MALDI-TOF with antimicrobial stewardship was found to save USD 29,205 per QALY and prevent one death per 14 patients tested compared to conventional methods without stewardship, with similar favorable incremental cost-effectiveness ratios for other rapid platforms [1]. Molecular rapid diagnostics in the emergency department for severe sepsis and septic shock were cost-saving, with incremental cost-effectiveness ratios as low as USD 7302 per death averted, depending on assay cost and reduction in length of stay [23,24].

Reduction in hospital length of stay is a consistent outcome, with rapid diagnostics plus stewardship reducing length of stay by 2–2.5 days compared to conventional methods [17,18]. This translates into substantial hospital cost savings, including lower total costs per bloodstream infection and reduced ICU costs [18].

Avoided antimicrobial costs are achieved through earlier de-escalation and targeted therapy, with the contemporary evidence base indicating decreased inappropriate antimicrobial use and overall antibiotic days [1,18]. Avoided ICU admissions and reduced ICU costs are reported, as earlier appropriate therapy can prevent clinical deterioration and shorten ICU stays [18].

Impact on antimicrobial resistance is addressed by facilitating timely de-escalation and reducing unnecessary broad-spectrum antibiotic exposure, which may help curb resistance rates over time [1,2].

These outcomes are most pronounced when rapid diagnostics are combined with antimicrobial stewardship programs, as supported by the American Society for Microbiology guidelines [12].

## 4. Discussion 

To contextualize these findings, the following discussion is structured around the main domains identified in the Results section, beginning with differences between diagnostic modalities.

### 4.1. Diagnostic Modalities and Comparative Performance (PCR Panels vs. MALDI-TOF vs. Combined Workflows)

The available evidence shows that PCR panels, MALDI-TOF, and combined diagnostic workflows provide clinically meaningful advantages in the diagnosis of bloodstream infections. However, their impact depends largely on how these technologies are integrated into diagnostic and therapeutic pathways.

PCR-based multiplex panels demonstrate high diagnostic accuracy, with a reported sensitivity of 92–99% and specificity of 99–100% for pathogen and resistance gene detection. They also offer rapid turnaround times (1–4 h). Economic evaluations indicate that PCR panels can shorten time to optimal antimicrobial therapy and may reduce hospital length of stay and mortality, particularly when results are promptly translated into treatment modification [2,13,31]. Although associated with higher laboratory costs, these approaches may generate downstream savings through earlier therapeutic optimization and more efficient resource use [13].

A potential limitation of highly sensitive molecular diagnostics is the risk of overdiagnosis or overtreatment. Detection of microbial DNA or resistance markers does not always indicate clinically relevant infection, particularly in cases of contamination, colonization, or residual nucleic material from non-viable organisms. Without appropriate clinical interpretation, such findings may lead to unnecessary antimicrobial use. However, this risk can be mitigated through integration with antimicrobial stewardship programs, multidisciplinary review, and alignment with clinical presentation. In this context, rapid diagnostics should be considered decision-support tools requiring careful interpretation rather than standalone determinants of therapy.

MALDI-TOF also provides substantial clinical value through rapid species-level identification, typically within one hour after blood culture positivity. Its implementation has been associated with reduced time to effective therapy, lower mortality, and decreased hospitalization costs, with reported mean savings of approximately USD 4140 per patient [1,15]. Meta-analyses further suggest that MALDI-TOF is among the most economically favorable strategies, with estimated savings of up to USD 29,205 per quality-adjusted life year and one death averted per 14 patients tested [1,15]. However, randomized trials indicate that MALDI-TOF alone may not consistently demonstrate cost-effectiveness, highlighting the importance of integration with clinical decision-making processes [6].

From a clinical perspective, direct-from-blood molecular assays offer the potential advantage of bypassing blood culture incubation, thereby enabling earlier pathogen detection in selected patients, especially in severe sepsis or septic shock. This earlier access to microbiological information may shorten time to appropriate therapy compared with blood culture-based rapid diagnostics, which still depend on culture positivity before testing can begin. However, blood culture-based rapid methods remain more established in routine practice and are supported by a broader evidence base demonstrating improvements in therapeutic optimization, length of stay, and downstream outcomes. From an economic perspective, direct-from-blood approaches may be attractive in high-acuity settings where even small reductions in treatment delay can yield substantial benefit, but they are typically associated with higher assay costs and greater uncertainty regarding cost-effectiveness across settings. By contrast, blood culture-based rapid diagnostics are more consistently supported by available economic evaluations, particularly when implemented within stewardship-supported workflows and efficient laboratory systems.

An additional consideration is the difference in diagnostic performance between direct-from-blood assays and tests performed on positive blood cultures. Direct-from-blood approaches may demonstrate lower sensitivity and specificity, which can limit their effectiveness in routine use. However, in patients already receiving antimicrobial therapy, where blood culture sensitivity is reduced, these assays may still provide clinically relevant information by enabling earlier pathogen detection. From an economic perspective, this introduces a trade-off: lower diagnostic accuracy may lead to continued empirical therapy or uncertainty in decision-making, while earlier detection in selected high-risk patients may reduce delays in appropriate treatment and associated downstream costs. Consequently, the value of direct-from-blood diagnostics is likely to be context-dependent, with the greatest benefit in high-acuity settings and in populations with prior antibiotic exposure.

Across modalities, the greatest improvements in clinical and economic outcomes are observed when rapid diagnostics are embedded within coordinated workflows that include active communication and antimicrobial stewardship support. Network meta-analyses show that such integrated approaches reduce mortality, shorten hospital stay, and accelerate time to optimal therapy by 18–29 h compared with conventional pathways [2,12]. Accordingly, the American Society for Microbiology recommends combining rapid diagnostics with active communication to facilitate earlier targeted therapy and reduce hospitalization duration [12].

While differences between diagnostic modalities are important, their clinical and economic impact is strongly dependent on how diagnostic information is translated into therapeutic decisions, particularly through antimicrobial stewardship integration.

### 4.2. Role of Antimicrobial Stewardship Integration (ASPs)

A consistent finding across the literature is that the clinical and economic value of rapid microbiological diagnostics is maximized when implemented alongside antimicrobial stewardship programs (ASPs). Rapid diagnostic tests alone do not consistently improve mortality, hospital length of stay, or time to optimal therapy compared with conventional pathways. Similarly, stewardship activities without rapid diagnostic input are limited by delayed pathogen identification and susceptibility data. When combined, however, these elements act synergistically.

Integration of rapid diagnostic tests with ASP interventions is associated with reduced mortality, shorter hospital stays, and faster transitions to appropriate antimicrobial therapy, with improvements of up to 29 h in time to optimal treatment [2,17]. Economic analyses further show that strategies combining PCR panels or MALDI-TOF with stewardship programs are highly cost-effective and often dominate conventional diagnostic pathways in probabilistic models [1].

These findings are supported by professional guidelines. The American Society for Microbiology recommends rapid diagnostic testing with active communication between laboratory and clinical teams, while the Infectious Diseases Society of America and the Society for Healthcare Epidemiology of America emphasize real-time stewardship review to improve time to effective therapy, antimicrobial de-escalation, and patient outcomes [12,47]. Overall, the evidence indicates that rapid microbiological methods are most effective when implemented within integrated stewardship-informed care models rather than as standalone technologies [1,2,3,12,17,19,20,47].

**Figure 2:**
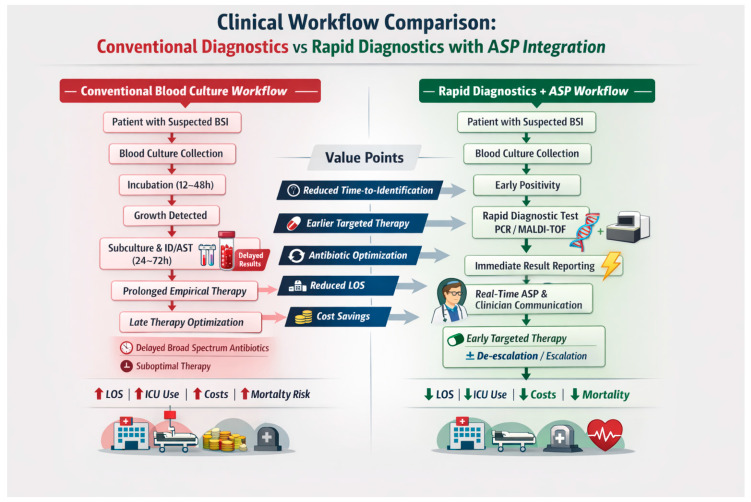
Clinical Workflow Comparison: Conventional Diagnostics vs. Rapid Diagnostics with ASP Integration.

To facilitate interpretation of these mechanisms, Figure 2 presents a schematic comparison of conventional diagnostic workflows and rapid diagnostic pathways integrated with antimicrobial stewardship and real-time communication.

An important conceptual consideration is that rapid diagnostic technologies should not be interpreted as standalone interventions. Rather, they function as components of a broader, multi-element care pathway that includes antimicrobial stewardship, real-time communication, and optimized clinical workflows. The observed improvements in clinical and economic outcomes are therefore unlikely to be attributable to the diagnostic platform alone. Instead, they reflect the combined and interdependent effects of timely pathogen identification, effective interpretation, and rapid therapeutic action.

Beyond stewardship integration, the effectiveness and value of rapid diagnostics are further shaped by the broader healthcare context in which they are implemented.

### 4.3. Contextual Factors and Healthcare Settings: High-Income vs. Resource-Limited Contexts

The effectiveness and economic value of rapid microbiological diagnostics vary across healthcare settings. In high-income environments, where laboratory capacity, clinical staffing, and stewardship infrastructure are well established, rapid diagnostics are consistently associated with improved clinical and economic outcomes. Technologies such as PCR panels and MALDI-TOF, particularly when integrated with stewardship programs, reduce mortality, shorten hospital length of stay, and decrease healthcare expenditures [1,2,14,17]. Under these conditions, favorable outcomes—including cost per life saved, cost per quality-adjusted life year gained, and avoided ICU and antimicrobial costs—are commonly reported [1,2,14,17].

In resource-limited settings, benefits are less consistent and strongly dependent on contextual factors such as laboratory infrastructure, blood culture turnaround times, resistance epidemiology, and assay pricing. Modeling studies suggest that molecular diagnostics can reduce mortality, hospital stay, and inappropriate antibiotic use, but only when diagnostic coverage is adequate, turnaround times are optimized, and per-test costs remain relatively low (ideally ≤ USD 100) [27]. Where laboratory capacity is limited, processing is delayed, or stewardship support is insufficient, these advantages may be substantially reduced [27,28]. Comparative studies further indicate that, although rapid methods improve turnaround time and therapy adequacy in high-income settings, their added value may be limited in high-resistance, resource-constrained environments lacking adequate infrastructure [28].

These findings indicate that adoption strategies for rapid microbiological diagnostics should be context-specific and account for local health system readiness, laboratory capacity, and antimicrobial resistance patterns. These contextual differences are closely linked to variations in cost structures and resource utilization, which are central to the economic evaluation of rapid diagnostic strategies.

### 4.4. Direct vs. Indirect Costs Considered

Economic evaluations of rapid microbiological diagnostics typically include both direct and indirect cost components. Direct costs encompass laboratory expenditures for diagnostic assays (e.g., PCR panels, MALDI-TOF), blood culture processing, antimicrobial therapy, and hospitalization, including ward and intensive care unit bed-days. These costs are influenced by assay pricing, per-day hospitalization costs, and length of stay, which may be reduced when rapid diagnostics enable earlier therapeutic adjustment [14,23,24,48].

Indirect costs reflect broader health system and societal impacts, including avoided mortality, gains in quality-adjusted life expectancy, reduced ICU utilization, and decreased exposure to inappropriate antimicrobial therapy. Economic models commonly incorporate outcomes such as QALYs gained and deaths averted, and may also include productivity gains from earlier recovery and discharge [14,23,24]. Integration of antimicrobial stewardship programs can further amplify these benefits by optimizing antimicrobial use and reducing unnecessary broad-spectrum therapy [1,2,17]. Considering both cost domains is essential because laboratory expenditures alone may underestimate the full economic value of rapid microbiological diagnostics when downstream clinical and health system impacts are taken into account. Building on these cost considerations, it is essential to examine the key mechanisms through which rapid diagnostics generate clinical and economic value.

### 4.5. Determinants of Clinical and Economic Outcomes

Several mechanisms underpin the cost-effectiveness of rapid microbiological diagnostics in bloodstream infection management.

Faster initiation of targeted therapy is a primary driver. Rapid diagnostics combined with stewardship programs reduce time to optimal antimicrobial therapy by approximately 18–29 h compared with conventional pathways [2,49]. Earlier therapeutic adjustment can lower mortality, shorten hospital stay, and improve healthcare resource utilization.

De-escalation and reduced use of broad-spectrum antibiotics represent another key mechanism. Early pathogen identification enables faster transition from empirical to targeted therapy, reducing unnecessary antimicrobial exposure and associated costs, while contributing to antimicrobial resistance containment [47,50]. Studies consistently report reductions in broad-spectrum antibiotic use and increased targeted therapy when rapid diagnostics are integrated with stewardship programs [19,49,51].

Prevention of severe complications, including progression to septic shock, also contributes to cost-effectiveness. Earlier identification and optimized therapy reduce inappropriate treatment, a major determinant of adverse outcomes in sepsis. By preventing complications requiring intensive care, rapid diagnostics may reduce ICU utilization and overall hospital costs [2,11,23].

Together, these mechanisms explain how rapid microbiological diagnostics improve outcomes while reducing healthcare expenditures when implemented within coordinated, stewardship-supported pathways [1,2,47,52]. These findings have direct clinical implications, as they support the integration of rapid diagnostics into routine decision-making processes to enable earlier targeted therapy and more efficient use of healthcare resources, with corresponding relevance for health policy and system-level planning.

However, the realization of these benefits in practice depends on multiple implementation factors that may facilitate or constrain their impact.

### 4.6. Implementation Barriers and Contextual Factors

Despite favorable downstream outcomes, implementation remains challenging. Upfront capital requirements are substantial and include acquisition of specialized platforms (e.g., MALDI-TOF, PCR systems), laboratory adaptation, test validation, and staff training. In many hospitals, these costs are not directly reimbursed, as rapid testing is often absorbed within broader laboratory budgets [6,18].

Operational costs add further complexity, including reagents, consumables, maintenance, and personnel required not only to perform testing but also to ensure real-time interpretation and clinical action. The availability of pharmacy or stewardship staff is critical, as the value of rapid diagnostics diminishes when results are not promptly translated into therapeutic decisions [3]. Limited staffing and non-continuous laboratory services can erode time advantages and reduce cost-effectiveness [30].

Economic evaluations show that savings depend not only on the diagnostic platform but also on workflow efficiency, institutional throughput, and the ability to convert information into action [1,6,14,18,30]. Accordingly, implementation decisions should consider local infrastructure and organizational readiness in addition to published cost-effectiveness estimates [12].

Among these factors, laboratory workflow integration plays a particularly critical role in determining whether the theoretical advantages of rapid diagnostics are achieved in real-world settings.

### 4.7. Laboratory Workflow Integration

Laboratory workflow integration is a key determinant of real-world effectiveness. Although rapid diagnostics may have excellent analytical performance, their impact is reduced when pre-analytical, analytical, or post-analytical processes introduce delays. A major barrier is limited laboratory operating time. In many institutions, blood cultures and rapid diagnostic procedures are not processed continuously, and result validation is restricted to standard daytime schedules. European data indicate that only a minority of laboratories provide round-the-clock incubation, processing, and reporting for positive blood cultures [22,30]. Even when rapid assays are available, preparation delays, batching, and personnel constraints can consume a substantial proportion of the theoretical turnaround-time advantage [30].

Effective implementation therefore requires optimization across the entire workflow, including timely sample collection and transport, immediate incubation and positivity handling, rapid identification and susceptibility testing, and direct communication of actionable results to clinical teams and stewardship personnel [3,5]. Protocols, staff training, and pathway redesign are often necessary to achieve this level of performance [5,12,52,53]. Without such alignment, rapid diagnostic technologies may underperform relative to their expected clinical and economic potential.

In parallel with workflow considerations, the capacity and organization of antimicrobial stewardship programs remain key determinants of effective implementation.

### 4.8. ASP Capacity and Multidisciplinary Coordination

The performance of stewardship-linked rapid diagnostics depends on ASP capacity and multidisciplinary coordination. Many hospitals lack sufficient infectious disease–trained pharmacists and physicians, limiting the feasibility of real-time review and intervention [54]. In such settings, stewardship responsibilities may be delegated to non-specialists, potentially reducing the consistency and quality of interpretation.

Knowledge gaps regarding the capabilities and limitations of rapid platforms can further complicate decision-making. Discordant findings between rapid and conventional methods may lead to inappropriate recommendations if microbiology, pharmacy, and clinical teams are not adequately aligned [51]. Rapid diagnostics therefore require more than technology adoption; they depend on structured communication models in which laboratory staff, stewardship teams, and clinicians operate within a shared interpretive framework [51,52,55].

Structured communication protocols, ongoing education, and diagnostic stewardship subgroups can strengthen this integration [55,56]. Current IDSA and SHEA guidance emphasizes that real-time ASP involvement and rapid result notification are essential for improving time to effective therapy and antimicrobial optimization [47]. Consequently, insufficient ASP capacity can substantially limit the real-world value of rapid diagnostics.

These system-level factors are closely related to challenges in clinical adoption, including the need for appropriate training and alignment between diagnostic output and clinical decision-making.

### 4.9. Clinical Adoption Challenges and Training Requirements

Clinical adoption is influenced by training and implementation readiness. Laboratory personnel must be competent in the operation, validation, and troubleshooting of platforms such as MALDI-TOF and multiplex PCR. At the same time, clinicians and stewardship teams need to understand the capabilities and limitations of these tests, including interpretation of resistance markers, potential discordance with standard culture, and implications for treatment modification [12,52,57].

Without adequate training, rapid results may be underused, misinterpreted, or acted upon too slowly to confer clinical benefit. Institutions therefore require not only initial implementation training but also ongoing competency assessment, protocol refinement, and diagnostic stewardship processes responsive to local epidemiology and evolving resistance patterns [25,52]. Training is thus an integral component of value realization rather than an auxiliary consideration.

At a broader level, these implementation challenges are embedded within wider health system characteristics, including reimbursement structures, pricing, and regional priorities.

### 4.10. Health System Heterogeneity (Reimbursement, Pricing, Regional Priorities)

Health system heterogeneity has important implications for implementation and scale-up. Reimbursement mechanisms vary widely across countries and even between hospitals within the same system. In many settings, rapid microbiological tests are not reimbursed separately, weakening financial incentives for adoption, particularly in smaller institutions or those with constrained budgets [36].

Local pricing is also a key factor. Costs for platforms, consumables, maintenance, and personnel differ across regions, limiting the transferability of published cost-effectiveness results without adjustment for local procurement conditions or test volumes [1,36]. Regional priorities further shape adoption. High-income systems may emphasize mortality reduction, shorter hospital stay, and resistance containment, whereas resource-limited settings may prioritize basic laboratory capacity and immediate cost control [1,9]. Local epidemiology, resistance patterns, and system organization therefore influence which strategies are most appropriate and economically justified [5,25].

These variations have direct implications for policy and decision-making across different stakeholder perspectives.

### 4.11. Policy and Practice Implications

#### 4.11.1. Decision-Making Perspectives (Payer, Hospital, Societal)

The implications of rapid microbiological diagnostics vary by decision-making perspective. From the payer perspective, these technologies are generally supported as cost-effective, particularly when combined with ASPs, as they reduce mortality, shorten hospital stay, and lower overall healthcare expenditures [1,2]. This supports reimbursement models that recognize value beyond assay costs alone.

From the hospital perspective, the key consideration is whether upfront and operational costs are offset by improved outcomes and reduced downstream expenditure. Evidence suggests this balance is most favorable when rapid diagnostics are embedded within workflows enabling timely therapeutic response, real-time communication, and stewardship involvement [3,12,52].

From the societal perspective, rapid diagnostics contribute to value-based care by improving outcomes, reducing inappropriate antimicrobial use, and supporting antimicrobial resistance control. Their impact therefore extends beyond individual cases and aligns with broader public health priorities [2,12,47].

#### 4.11.2. Value-Based Diagnostics

The concept of value-based diagnostics is particularly relevant in bloodstream infection management, where value derives not only from diagnostic accuracy but also from the ability to influence downstream care. Rapid microbiological methods create value by enabling earlier, more precise, and more efficient treatment decisions, improving outcomes relative to resources invested. Accordingly, diagnostics should be evaluated not only on analytical performance or laboratory cost, but also on their contribution to clinically meaningful outcomes and health system efficiency [1,2].

For hospitals and payers, this implies that investment decisions should consider avoided mortality, reduced hospitalization, lower ICU utilization, and more appropriate antimicrobial use. More broadly, value-based implementation requires that technology adoption be accompanied by stewardship capacity, effective communication pathways, and workflow integration [12,52]. Without these supporting elements, the potential value of rapid diagnostics may not be realized in practice.

In light of these considerations, several practical recommendations can be formulated to support wider and more effective adoption.

### 4.12. Recommendations for Wider Adoption

Wider adoption of rapid microbiological diagnostics should be pursued through coordinated policy and institutional strategies. First, reimbursement and funding mechanisms should better reflect the broader clinical and economic value of these technologies, particularly when used in stewardship-supported care models [1,2]. Second, hospitals should prioritize implementation models that include real-time communication, laboratory–clinical coordination, and adequate ASP capacity rather than focusing only on test procurement [12,52].

Third, laboratory infrastructure and workforce development should be strengthened to support timely sample processing, result reporting, and interpretation. Fourth, training should be treated as a strategic implementation priority for laboratory staff, pharmacists, infectious disease specialists, and frontline clinicians. Finally, rapid diagnostics should be incorporated into national guidance, stewardship frameworks, and quality improvement initiatives as part of a broader effort to optimize bloodstream infection management and reduce antimicrobial resistance [2,12,52].

Beyond institutional implementation, rapid diagnostics also have an important role within broader antimicrobial resistance strategies.

### 4.13. Integration into National Antimicrobial Resistance Strategies

At the policy level, rapid microbiological diagnostics should be integrated into national antimicrobial resistance strategies as tools for earlier targeted treatment and more effective stewardship. As the strongest evidence supports their use in combination with ASPs, policies should promote both technology uptake and stewardship capacity building rather than addressing these components in isolation [1,2,12].

Implementation also requires attention to reimbursement and equitable access. National guidance should support adoption in high-burden settings, promote standardized protocols for result interpretation and intervention, and reduce regional disparities in access to diagnostic innovation [1,27]. Integration into national surveillance and quality improvement programs may further strengthen monitoring of antimicrobial use, resistance trends, and patient outcomes [2,12,27].

Despite these promising implications, the interpretation of current evidence is subject to several important limitations.

### 4.14. Limitations of the Current Evidence Base

This review has several limitations inherent to its narrative design. Although a structured literature search and selection process was applied, the review does not follow a fully systematic review or meta-analytic framework. As a result, the synthesis may be subject to selection bias, variability in study inclusion, and heterogeneity in study design, populations, and outcome reporting. In addition, the absence of formal quantitative synthesis limits the ability to directly compare results across studies or to generate pooled estimates of clinical and economic effects. Nevertheless, the narrative approach allows for a broader integration of clinical, economic, and implementation perspectives, which is particularly relevant in a heterogeneous and rapidly evolving field.

The current evidence base also presents several important limitations. First, there is substantial heterogeneity in study design, patient populations, diagnostic platforms, comparator strategies, outcome definitions, and cost inputs. This complicates cross-study comparison and limits the transferability of economic conclusions between settings [1,2,32]. In some analyses, costs are modeled or estimated rather than derived from actual expenditure data, which may further reduce generalizability. Studies with more comprehensive modeling frameworks and sensitivity analyses tend to report more stable estimates of cost-effectiveness, whereas analyses with limited time horizons or simplified assumptions may overestimate or underestimate long-term economic value.

Second, much of the literature is retrospective, single-center, or quasi-experimental, with relatively few large prospective, multicenter, or pragmatic real-world studies [12,42,46,58]. This limits the strength of inference regarding effectiveness and cost-effectiveness across diverse care settings.

Third, ASP integration is inconsistent across studies. Some investigations evaluate rapid diagnostics alone, while others assess combined diagnostic–stewardship interventions with varying intensity and structure [1,2,52,58]. In parallel, most studies examine bundled strategies, making it difficult to disentangle the independent contribution of the diagnostic platform from the synergistic effects of stewardship, communication, and workflow integration. This limitation is particularly relevant for economic interpretation, as cost-effectiveness may depend heavily on system-level factors.

Fourth, many economic evaluations use short-term horizons focused on in-hospital outcomes. This likely underestimates the broader value of rapid diagnostics by failing to capture longer-term effects such as readmissions, resistance dynamics, and downstream healthcare savings [1,46,58].

A further limitation relates to cross-study comparability. The included studies originate from diverse healthcare systems with substantial variation in reimbursement structures, unit costs, assay pricing, and willingness-to-pay thresholds. Differences in currency year, inflation adjustments, and costing methodologies further limit direct comparison. As a result, economic outcomes such as cost per QALY gained or total cost savings should be interpreted as context-specific rather than universally generalizable estimates. This limitation is inherent to narrative syntheses in heterogeneous economic literature and underscores the need for standardized reporting and harmonized methodological frameworks.

In addition, variability in methodological quality across included studies may influence the robustness of the conclusions. While a structured qualitative appraisal was conducted using CHEERS 2022 domains and the Philips checklist, no formal quality scoring or exclusion based on predefined thresholds was applied. Consequently, findings reflect patterns of consistency rather than a quality-weighted synthesis and should be interpreted with consideration of underlying methodological differences.

Another limitation of the present review is the emphasis on PCR-based diagnostics and MALDI-TOF platforms, which may not fully capture the diversity of emerging rapid diagnostic technologies. Methods such as loop-mediated isothermal amplification (LAMP), microarray-based assays, and sequencing-based approaches are increasingly being developed and evaluated. However, these technologies remain underrepresented in clinical and economic studies, particularly in the context of bloodstream infections and antimicrobial stewardship integration, and their potential value may therefore be underrepresented in this synthesis.

### 4.15. Future Research Directions

Several research priorities emerge from the current evidence gaps. First, there is a clear need to standardize economic evaluation methods, including the definition of cost categories, outcome measures, and analytic perspectives, in order to facilitate more robust comparison across studies and settings [1,32]. The use of more harmonized frameworks may improve the quality and interpretability of future evidence [12,32].

Second, more prospective multicenter research is needed. Future studies should include diverse patient populations, hospital types, and stewardship models to improve generalizability and better reflect real-world implementation conditions [2,7,9,12].

Third, future evaluations should extend beyond short-term hospital outcomes and examine longer-term consequences, especially the impact on antimicrobial resistance, readmissions, and downstream healthcare utilization. Although modeling studies suggest that rapid diagnostics may reduce inappropriate antibiotic use and antimicrobial resistance, stronger prospective evidence is needed [27,59].

Fourth, additional research is necessary in low- and middle-income countries and across heterogeneous hospital settings. These studies should explicitly address local infrastructure, affordability, stewardship capacity, and scalability in order to identify context-appropriate adoption strategies [8,27,59].

Finally, emerging technologies, including isothermal amplification methods, microarray-based diagnostics, next-generation sequencing, and artificial intelligence-supported diagnostic platforms, warrant dedicated evaluation in terms of both clinical effectiveness and economic impact. As these approaches transition from experimental settings to routine clinical use, robust evidence will be needed to determine their comparative value, implementation requirements, and cost-effectiveness across different healthcare contexts [25,29,59].

Overall, addressing these gaps will help define more precisely how rapid microbiological diagnostics can be implemented in an equitable, efficient, and value-based manner for the management of bloodstream infections.

## 5. Conclusions

Rapid microbiological diagnostics, when embedded within stewardship-supported and well-integrated clinical workflows, represent a significant advancement in the management of bloodstream infections, delivering both clinical and economic benefits. Evidence consistently shows that technologies such as PCR-based multiplex panels and MALDI-TOF mass spectrometry reduce time to pathogen identification and optimal antimicrobial therapy, leading to shorter hospital stays, lower intensive care utilization, and reduced healthcare costs.

The available economic evidence supports investment in these technologies, as downstream savings and improved outcomes often offset initial implementation costs. In addition to direct economic benefits, rapid diagnostics contribute to public health goals by enabling earlier targeted therapy, reducing unnecessary antimicrobial use, and supporting antimicrobial resistance control.

However, their value remains highly context-dependent. Laboratory infrastructure, stewardship capacity, clinical workflows, reimbursement structures, and local epidemiology all influence effectiveness and cost-effectiveness. Accordingly, adoption strategies should be tailored to specific health system contexts and supported by coordinated investments in diagnostic capacity, stewardship programs, and clinical integration.

Overall, rapid microbiological diagnostics are a key component of value-based bloodstream infection management, with the potential to improve patient outcomes, optimize antimicrobial use, and enhance healthcare system sustainability when appropriately implemented.

## Figures and Tables

**Table 2:** Summary of economic outcomes associated with rapid microbiological diagnostic modalities for bloodstream infections.

Diagnostic Modality	Typical Turnaround Time	Economic Evaluation Types	Key Economic Outcomes Reported	Cost-Effectiveness Conclusion	Key Modifiers of Value
MALDI-TOF (±ASP)	30–60 min after BC positivity	CEA, CUA, cost-consequence	Cost per QALY gained; cost per life saved; reduced LOS; avoided ICU days	Cost-effective/cost-saving when combined with ASP; inconsistent value without ASP	ASP integration; laboratory operating hours; LOS reduction magnitude
Multiplex PCR panels (e.g., BCID)	1–4 h	CEA, CUA, BIA	Favorable ICERs; reduced total hospital costs; avoided antimicrobial costs	Cost-effective, often cost-saving despite higher assay costs	Test price; speed of result communication; stewardship response
Direct-from-blood molecular assays	1–4 h (no culture delay)	CEA, modeling studies	Cost per death averted; reduced ICU utilization	Cost-effective in high-severity settings	Disease severity; prevalence of resistance; assay cost
Rapid phenotypic AST platforms	4–8 h after BC positivity	CEA, cost-consequence	Reduced LOS; ICU cost avoidance	Context-dependent; strongest with ASP	Workflow integration; timing vs. standard AST
Combined diagnostic workflows (RDT + ASP + communication)	Same-day actionable results	CEA, CUA, BIA	Consistently favorable ICERs; net cost savings	Highest economic value across settings	Stewardship capacity; staffing; real-time reporting
Conventional blood culture workflows	16–72 h	Comparator	Higher LOS; higher mortality-related costs	Reference strategy	—

Abbreviations: ASP, antimicrobial stewardship program; AST, antimicrobial susceptibility testing; BC, blood culture; BCID, blood culture identification; BIA, budget impact analysis; CEA, cost-effectiveness analysis; CUA, cost–utility analysis; ICER, incremental cost-effectiveness ratio; ICU, intensive care unit; LOS, length of stay; MALDI-TOF, matrix-assisted laser desorption/ionization time-of-flight; QALY, quality-adjusted life year; RDT, rapid diagnostic test.

## Data Availability

No new data were created or analyzed in this study. Data sharing is not applicable to this article.

## Abbreviations

The following abbreviations are used in this manuscript:

AMR	Antimicrobial resistance
ASP	Antimicrobial stewardship program
AST	Antimicrobial susceptibility testing
BC	Blood culture
BCID	Blood Culture Identification
BIA	Budget impact analysis
BSI	Bloodstream infection
CEA	Cost-effectiveness analysis
CHEERS	Consolidated Health Economic Evaluation Reporting Standards
CUA	Cost–utility analysis
dRAST	Direct Rapid Antimicrobial Susceptibility Test
DNA	Deoxyribonucleic acid
FLAT MS	Fast Lipid Analysis Technique Mass Spectrometry
ICER	Incremental cost-effectiveness ratio
ICU	Intensive care unit
ID	Identification
IDSA	Infectious Diseases Society of America
LAMP	Loop-mediated isothermal amplification
LMIC	Low- and middle-income country
LOS	Length of stay
MALDI-TOF	Matrix-assisted laser desorption/ionization time-of-flight
MEDLINE	Medical Literature Analysis and Retrieval System Online
PCR	Polymerase chain reaction
PRISMA	Preferred Reporting Items for Systematic Reviews and Meta-Analyses
QALY	Quality-adjusted life year
RDT	Rapid diagnostic test
RNA	Ribonucleic acid
SHEA	Society for Healthcare Epidemiology of America
T2MR	T2 Magnetic Resonance

## Appendix A

### Appendix A.1. Database Search Strategies

**Table A1:** Structured search strategies and outputs for PubMed, Scopus, and Web of Science.

Database	Search Strategy
PubMed (MEDLINE) with MeSH	(“bloodstream infections” [MeSH Terms] OR “sepsis” [MeSH Terms] OR “bacteremia” [MeSH Terms] OR “bloodstream infection” [tiab] OR “bacteremia” [tiab] OR “bacteraemia” [tiab]) AND (“diagnostic techniques and procedures” [MeSH Terms] OR “microbiological techniques” [MeSH Terms] OR “mass spectrometry” [MeSH Terms] OR “nucleic acid amplification techniques” [MeSH Terms] OR “rapid diagnostic” [tiab] OR “rapid identification” [tiab] OR “MALDI-TOF” [tiab] OR “multiplex PCR” [tiab] OR “rapid AST” [tiab]) AND (“cost-benefit analysis” [MeSH Terms] OR “economics” [MeSH Terms] OR “healthcare costs” [MeSH Terms] OR “economic evaluation” [tiab] OR “cost-effectiveness” [tiab] OR “cost-benefit” [tiab] OR “cost-utility” [tiab] OR “budget impact” [tiab] OR “healthcare costs” [tiab])
Scopus	TITLE-ABS-KEY (“bloodstream infection” OR bacteremia OR bacteraemia) AND TITLE-ABS-KEY (“rapid diagnostic” OR “rapid identification” OR “MALDI-TOF” OR “multiplex PCR” OR “rapid AST”) AND TITLE-ABS-KEY (“economic evaluation” OR “cost-effectiveness” OR “cost-benefit” OR “cost-utility” OR “budget impact” OR “healthcare costs”)
Web of Science	TS = (“bloodstream infection” OR bacteremia OR bacteraemia) AND TS = (“rapid diagnostic” OR “rapid identification” OR “MALDI-TOF” OR “multiplex PCR” OR “rapid AST”) AND TS = (“economic evaluation” OR “cost-effectiveness” OR “cost-benefit” OR “cost-utility” OR “budget impact” OR “healthcare costs”)
EconLit	AB(“bloodstream infection” OR bacteremia OR bacteraemia) AND AB(“rapid diagnostic” OR “MALDI-TOF” OR “multiplex PCR”) AND AB(“economic evaluation” OR “cost-effectiveness” OR “budget impact”)
Google Scholar	“bloodstream infection” “rapid diagnostics” “economic evaluation”
Additional filters for all databases:	
Language:	English
Timespan:	Last 15 years
Categories:	“Health Policy & Services,” “Microbiology,” “Medical Laboratory Technology,” “Pharmacoeconomics & Health Economics”

### Appendix A.2. PRISMA 2020 Checklist

**Table A2:** PRISMA 2020 checklist.

**Section and Topic**	**Item #**	**Checklist Item**	**Location Where Item is Reported**
TITLE			
Title	1	Identify the report as a systematic review.	N/A State-of-the-Art Evidence Review
ABSTRACT			
Abstract	2	See the PRISMA 2020 for Abstracts checklist.	p. 1
INTRODUCTION			
Rationale	3	Describe the rationale for the review in the context of existing knowledge.	Introduction, Section 1.4; p. 3
Objectives	4	Provide an explicit statement of the objective(s) or question(s) the review addresses.	Introduction, Section 1.4; p. 4
METHODS			
Eligibility criteria	5	Specify the inclusion and exclusion criteria for the review and how studies were grouped for the syntheses.	Materials and Methods, Section 2.2.; p. 5
Information sources	6	Specify all databases, registers, websites, organizations, reference lists and other sources searched or consulted to identify studies. Specify the date when each source was last searched or consulted.	Materials and Methods, Section 2.1.; p. 6
Search strategy	7	Present the full search strategies for all databases, registers and websites, including any filters and limits used.	Appendix A.1., Table 1; pp. 22–23
Selection process	8	Specify the methods used to decide whether a study met the inclusion criteria of the review, including how many reviewers screened each record and each report retrieved, whether they worked independently, and if applicable, details of automation tools used in the process.	Materials and Methods, Section 2.3 and Section 2.4; pp. 5–6
Data collection process	9	Specify the methods used to collect data from reports, including how many reviewers collected data from each report, whether they worked independently, any processes for obtaining or confirming data from study investigators, and if applicable, details of automation tools used in the process.	Materials and Methods, Section 2.3 and Section 2.4; pp. 5–6
Data items	10a	List and define all outcomes for which data were sought. Specify whether all results that were compatible with each outcome domain in each study were sought (e.g., for all measures, time points, analyses), and if not, the methods used to decide which results to collect.	Materials and Methods, Section 2.2 and Section 2.3; p. 5
	10b	List and define all other variables for which data were sought (e.g., participant and intervention characteristics, funding sources). Describe any assumptions made about any missing or unclear information.	Materials and Methods, Section 2.3; p. 5
Study risk of bias assessment	11	Specify the methods used to assess risk of bias in the included studies, including details of the tool(s) used, how many reviewers assessed each study and whether they worked independently, and if applicable, details of automation tools used in the process.	Not applicable (narrative synthesis with qualitative appraisal; see Section 2.4, p. 6)
Effect measures	12	Specify for each outcome the effect measure(s) (e.g., risk ratio, mean difference) used in the synthesis or presentation of results.	Not applicable (no quantitative synthesis performed)
Synthesis methods	13a	Describe the processes used to decide which studies were eligible for each synthesis (e.g., tabulating the study intervention characteristics and comparing against the planned groups for each synthesis (item #5)).	Materials and Methods, Section 2.5; p. 6
	13b	Describe any methods required to prepare the data for presentation or synthesis, such as handling of missing summary statistics, or data conversions.	Not applicable (no data transformation required)
	13c	Describe any methods used to tabulate or visually display results of individual studies and syntheses.	Table 1, p. 8 and Table 2; p. 11 Narrative synthesis (Results and Discussion), pp. 7–21
	13d	Describe any methods used to synthesize results and provide a rationale for the choice(s). If meta-analysis was performed, describe the model(s), method(s) to identify the presence and extent of statistical heterogeneity, and software package(s) used.	Materials and Methods, Section 2.5.; p. 6
	13e	Describe any methods used to explore possible causes of heterogeneity among study results (e.g., subgroup analysis, meta-regression).	Not applicable (no formal heterogeneity analysis)
	13f	Describe any sensitivity analyses conducted to assess robustness of the synthesized results.	Not applicable (no sensitivity analysis performed)
Reporting bias assessment	14	Describe any methods used to assess risk of bias due to missing results in a synthesis (arising from reporting biases).	Not applicable
Certainty assessment	15	Describe any methods used to assess certainty (or confidence) in the body of evidence for an outcome.	Not applicable
RESULTS			
Study selection	16a	Describe the results of the search and selection process, from the number of records identified in the search to the number of studies included in the review, ideally using a flow diagram.	Results, pp. 7–12 Figure 1 (PRISMA diagram), p. 8
	16b	Cite studies that might appear to meet the inclusion criteria but were excluded, and explain why they were excluded.	Results (reasons for exclusion described), pp. 7
Study characteristics	17	Cite each included study and present its characteristics.	Results, pp. 7–12 Table 1, p. 8 and Table 2, p. 11
Risk of bias in studies	18	Present assessments of risk of bias for each included study.	Not applicable (qualitative appraisal only; see Section 2.4, p. 6)
Results of individual studies	19	For all outcomes, present, for each study, (a) summary statistics for each group (where appropriate) and (b) an effect estimate and its precision (e.g., confidence/credible interval), ideally using structured tables or plots.	Narrative synthesis (Results, pp. 7–12)
Results of syntheses	20a	For each synthesis, briefly summarize the characteristics and risk of bias among contributing studies.	Narrative synthesis (Results and Discussion), pp. 7–21
	20b	Present results of all statistical syntheses conducted. If meta-analysis was done, present for each the summary estimate and its precision (e.g., confidence/credible interval) and measures of statistical heterogeneity. If comparing groups, describe the direction of the effect.	Not applicable (no meta-analysis)
	20c	Present results of all investigations of possible causes of heterogeneity among study results.	Narrative discussion of heterogeneity (Results, pp. 7–12)
	20d	Present results of all sensitivity analyses conducted to assess the robustness of the synthesized results.	Not applicable
Reporting biases	21	Present assessments of risk of bias due to missing results (arising from reporting biases) for each synthesis assessed.	Not applicable (no formal assessment of reporting bias due to missing results was conducted because no quantitative synthesis or meta-analysis was performed)
Certainty of evidence	22	Present assessments of certainty (or confidence) in the body of evidence for each outcome assessed.	Not applicable (no formal certainty/confidence assessment framework, such as GRADE, was applied due to the narrative design and absence of pooled outcome estimates)
DISCUSSION			
Discussion	23a	Provide a general interpretation of the results in the context of other evidence.	Discussion, pp. 12–21
	23b	Discuss any limitations of the evidence included in the review.	Discussion, Section 4.14; pp. 19–20
	23c	Discuss any limitations of the review processes used.	Discussion, Section 4.14; pp. 19–20
	23d	Discuss implications of the results for practice, policy, and future research.	Discussion, Section 4.11, Section 4.12 and Section 4.13; pp. 18–19
OTHER INFORMATION			
Registration and protocol	24a	Provide registration information for the review, including register name and registration number, or state that the review was not registered.	Not registered
	24b	Indicate where the review protocol can be accessed, or state that a protocol was not prepared.	No protocol prepared
	24c	Describe and explain any amendments to information provided at registration or in the protocol.	Not applicable
Support	25	Describe sources of financial or non-financial support for the review, and the role of the funders or sponsors in the review.	This study is financed by the European Union—NextGenerationEU, through the National Recovery and Resilience Plan of the Republic of Bulgaria (project No. BG-RRP-2.004-0007-C01). The funder had no role in the design of the study, data collection, analysis, interpretation of data, or writing of the manuscript.
Competing interests	26	Declare any competing interests of review authors.	The authors declare no conflicts of interest.
Availability of data, code and other materials	27	Report which of the following are publicly available and where they can be found: template data collection forms; data extracted from included studies; data used for all analyses; analytic code; any other materials used in the review.	Research data can be obtained upon request.
